# Microvesicles isolated from 5-azacytidine-and-resveratrol-treated mesenchymal stem cells for the treatment of suspensory ligament injury in horse—a case report

**DOI:** 10.1186/s13287-019-1469-5

**Published:** 2019-12-18

**Authors:** Katarzyna Kornicka-Garbowska, Rafał Pędziwiatr, Paulina Woźniak, Katarzyna Kucharczyk, Krzysztof Marycz

**Affiliations:** 1 Department of Experimental Biology, Wroclaw University of Environmental and Life Sciences, Norwida 27B street, A7 building, 50-375 Wroclaw, Poland; 2 International Institute of Translational Medicine, Malin, Jesionowa 11, 55-114 Wisznia Mała, Poland; 3 EQUI-VET Clinic for Horses, Stogniowice 55A, 32-100 Proszowice, Poland; 4 Faculty of Veterinary Medicine, Equine Clinic-Equine Surgery, Justus-Liebig-University, 35392 Giessen, Germany

**Keywords:** Microvesicles, Suspensory ligament, Horse, Injury

## Abstract

**Background:**

In athlete horses, suspensory ligament (SL) injuries are the most common cause of lameness. Healing of SL injury is still problematic, and even proper rehabilitation and pharmacological therapy do not guarantee returning to the initial performance level. In our previous studies, we have shown that a combination of 5-azacytidine (AZA) and resveratrol (RES) exerts beneficial, rejuvenating effects on metabolic syndrome derived adipose-derived stem cells (ASCs). Thus, in the presented research, we investigate whether not only rejuvenated ASC but also microvesicles (MVs_AZA/RES_) secreted by them possess enhanced regenerative properties in SL injury.

**Methods:**

In the presented study, a 6-year-old Dutch Warmblood gelding, working in jumping, was diagnosed with SL injury using ultrasonography, Doppler, real-time elastography and thermography. As a therapeutic strategy, the affected animal was treated with extracellular microvesicles derived from ASC treated with the combination of 5-azacytydine (AZA) and resveratrol (RES) (MVs_AZA/RES_)_._

**Results:**

First, anti-apoptotic effects of MVs_AZA/RES_ were tested in co-culture with metabolic syndrome derived ASC. The proliferation of cells and expression of pro-apoptotic genes were investigated. Then, MVs_AZA/RES_ were injected directly into the injured SL of the Dutch Warmblood gelding. In vitro assays revealed that MVs_AZA/RES_ enhance the proliferation of ASC and exert an anti-apoptotic effect. In the affected horse, the application of MVs_AZA/RES_ resulted in increased lesion filling and improvement of angiogenesis and elasticity in injured tissue.

**Conclusions:**

As MVs_AZA/RES_ mimic several of the biological actions exerted by ASC, they have become an alternative for stem cell-based therapies and can be effectively applied for the treatment of SL injury in horses.

## Background

Musculoskeletal injuries are especially common in human athletes who train sprint or jumping [1]. Similar phenomenon occurs in sport horses as they are at substantial risk for impact injuries or overexertion. In consequence, local muscle or tendon trauma occurs leading to poor performance and lameness. In horses, muscle tissue represents over 50% of bodyweight; thus, its demand for energy and cardiac output during exercise is high [2]. Factors predisposing for injuries to list are pre-existing lameness or injury, early season competition and lack of warm-up. Among diagnostic techniques, ultrasonography (USG) is recognized as a useful imaging procedure [3] in which muscle or tendon enlargement and loss of normal echogenicity consistent with fibre disruption can be noticed in acute injuries. Another non-invasive procedure is thermography which enables the pictorial representation of the surface temperature of an object allowing for visualization of inflammatory changes [4]. Additionally, elastography-ultrasound technique allows to detect and measure tissue strain, providing valuable information regarding equine tendon, muscle and ligament injuries [5]. Repeated injury to certain muscle group may result in fibrosis and ossification, finally contributing to mechanical lameness. Horses affected with strains, depending on its severity, are provided with anti-inflammatory, muscle relaxants and non-steroidal drugs while exercise activity is reduced from days to months [6]. Among injuries, suspensory ligament (SL) damage in horses is the most common cause of lameness especially in athletic individuals in competing dressage. Ligament sprain leads to swelling, pain and heat. Interestingly, usually, the proximal aspect of the SL is affected [7]. Healing of SL injury is still problematic, and even proper rehabilitation does not guarantee returning to the initial performance level. Tendon is a tissue characterized by limited regeneration caused by the formation of scar tissue resulting from relatively low number of resident cells in relation to amount of matrix. One of the therapeutic approaches for tendon injuries is conservative management which includes cold applications, controlled exercises, eccentric tendon training and extracorporeal shockwave therapy [8]. In case of severe injuries, surgical therapy (fasciotomy) is recommended. New approaches are focused on the regenerative medicine and its tools. Application of platelet-rich plasma (PRP) and stem cell therapies is more and more common [9, 10].

Nowadays, mesenchymal stem cells (MSCs) are widely applied in both human and veterinary medicine. Due to their unique properties, e.g. multilineage differentiation and immunomodulatory action, MSCs have afforded great promise in the treatment of numerous diseases [11]. There are 344 registered clinical trials in different clinical trial phases aimed at investigating the potential of MSC therapy worldwide [12]. Since now, MSCs have been proved to be effective in the treatment of tissue injury, immune and musculoskeletal diseases and neurodegenerative disorders [13–15]. Although MSCs have a tendency to home to damaged tissue sites, their therapeutic mechanism probably is other than differentiation in particular cell type. It was shown that MSC transplantation promoted the regeneration of skeletal muscle in a rat injury model; however, cells did not differentiate into myofibers indicating that distinct mechanism is responsible for therapeutic outcome [16].

Accumulating body of evidence has revealed that therapeutic benefits of MSC mainly depend on their ability to secrete a wide range of trophic factors. It was demonstrated that the paracrine activity of those cells can be utilized in the treatment of multiple disorders including liver, kidney, lung and myocardial injuries [13]. It was showed that MSC secrete a wide range of growth factors including vascular endothelial growth factor (VEGF), basic fibroblast growth factors (bFGF), hepatocyte growth factor (HGF), insulin-like growth factor 1 (IGF-1), interleukin 6 (IL-6) and chemokine (C-C motif) ligand 2 (CCL-2) [17]. Most of them act as key mediators in angiogenesis, regeneration and prevention of cell apoptosis. These proteins are secreted as a cargo in extracellular microvesicles (MVs) and exosomes. MVs are membrane-covered vesicles of various shapes with a diameter varying between 50 to 1000 nm and more, formed by budding from the plasma membrane [18]. What is more, not only cytokines and growth factors can be transferred within MVs but also messenger RNA (mRNA), lipids, ribosomal RNA, siRNA and microRNA (miRNA) [12]. Pro-regenerative functions of MVs were proved in several tissues, including the kidney, heart, liver, nervous tissues, lung and muscles [19]. Obtained results indicated that MV functions are similar to those of MSCs—they improve the regeneration process, suppress inflammation and modulate immune response. Nowadays, MVs are of great interest in the scope of regenerative medicine as they can pave the way for the development of cell-free therapies. Currently, the most common method of MV manufacturing in the laboratory is ultracentrifugation of MSC culture medium [20]. In order to obtain a great amount of MVs, cells are cultured under certain distress as MVs are released with their cargo to combat stressful condition [21]. Interestingly, MV cargo can be modulated by the application of certain stress-inducing factor [22]. What is more, MV content strongly depends on cytophysiological properties of MSC they are obtained from [23–25]. Our previous studies have shown that a combination of 5-azacytydine (AZA) and resveratrol (RES) reverses aged phenotype of MSC isolated from equine metabolic syndrome diagnosed animals (MSC-EMS) [26–28]. In consequence, those rejuvenated cells were characterized by an increased proliferation rate, reduced apoptosis and enhanced synthesis of MVs. Furthermore, AZA/RES diminished oxidative stress and improved mitochondrial condition and dynamics in those cells reversing degenerative changes caused by EMS-associated systemic inflammation [27, 29]. In this report, we decided to analyse the properties of MVs isolated from MSC-EMS and test whether the combination of AZA/RES may become a factor able to enrich MV cargo with pro-regenerative proteins. As AZA/RES strongly modulates metabolism of MSC, we hypothesized that naturally it also regulates the amount and cargo of secreted MVs.

Since now, MVs have shown much promise and benefits—due to their physiochemical stability in the body, non-immunogenic character and unique cargo, they can become alternative for stem cell-based therapies [30]. It was proved that MSC-derived exosomes accelerate muscle regeneration via promotion of myogenesis and angiogenesis, mediated by miRNAs (e.g. miR-494) [31], which supports their application in orthopaedics. In the presented case, taking into consideration the beneficial effects of AZA/RES on aged MSC, we decided to investigate the clinical utility of MVs derived from MSC-EMS treated with these substances. For that reason, MVs were injected twice locally into injured suspensory ligament of a horse athlete.

## Methods

All reagents used in the study were purchased from Sigma Aldrich Poland unless indicated otherwise.

### Isolation and culture of ASC

Horses from which adipose tissue was collected were characterized in our previous study [32]. Animals from which samples were collected were divided into two groups: diagnosed with equine metabolic syndrome (EMS) and healthy (CTRL). ASCs were isolated in accordance with the procedure described before [29]. Briefly, adipose tissue was minced and incubated with 1 mg/ml solution of collagenase type I. After centrifugation, the remaining pellet was re-suspended in culture media—Dulbecco’s modified Eagle’s medium (DMEM) low glucose supplemented with 10% of foetal bovine serum (FBS) and 1% of penicillin-streptomycin (PS) solution. The media were changed every 2 days. After reaching 90% confluence, cells were passaged with TrypleExpress (Life Technologies). In order to isolate MVs, cells were passaged three times. When cells reach 80% confluence, cells were cultured in the presence of 0.5 μM of AZA and 0.05 μM of RES. After 24 h, the medium supplemented with AZA/RES was exchanged for serum-free DMEM with 1% PS. After 24 h, serum-free medium was collected for MV isolation.

### MV isolation

Serum-free medium was collected from AZA/RES-treated ASC isolated from EMS individuals, in order to isolate MVs. MVs were harvested in accordance with the protocol described previously by Szatanek et al. [33]—differential centrifugation/ultracentrifugation. Briefly, the medium was centrifuged at 300×*g* for 10 min, 2000×*g* for 10 min and 10,000×*g* for 30 min respectively. After each centrifugation, the supernatant is transferred to a new tube while the pellet discarded. The pellet from the last centrifugation was re-suspended in sterile Hank’s balanced salt solution (HBSS) as it consists of MV fraction. Those MVs were applied in in vitro and in vivo part of the study and are described in the figures as MVs_AZA/RES_.

### Evaluation of cellular proliferation

Growth kinetics of ASCs was examined using a resazurin assay kit (TOX8), following the manufacturer’s instructions as previously described [27]. To perform the assay, cells were seeded in 24-well plates at an initial concentration of 2 × 10^4^ per well. The next day, after cells attached, different concentrations of as MVs_AZA/RES_ were added to the wells. After 24 h of culture, the medium was exchanged for DMEM low glucose supplemented with 10% of TOX8, and after 120 min of incubation with the dye, the first measurement was performed. The absorbance of the supernatants was measured at a wavelength of 600 nm for resazurin, and 690 nm reference wavelength (Epoch, BioTek). Measurements were performed after 24, 48, 72 and 96 h of culture. DNA synthesis was investigated by measuring the incorporation of 5-bromo-2-deoxyuridine (BrdU) into cellular DNA with BrdU Cell Proliferation ELISA Kit (Abcam) in accordance with the manufacturer’s protocol. In this experiment, cells were pre-treated with 25 μg/ml MVs_AZA/RES_ for 24 h. Next, cells were incubated with BrdU overnight at 37 °C. The incorporation of BrdU was evaluated by incubation with anti-BrdU monoclonal antibody. Colour reaction was developed using 3,3,5,5-tetramethylbenzidine (TMB). Signal intensity was measured at a wavelength of 450/550 nm (Epoch; BioTek).

### TUNEL staining

To perform the assay, cells were pre-treated with 25 μg/ml MVs_AZA/RES_ for 24 h. DNA fragmentation was detected using TUNEL Assay Kit-BrdU-Red (Abcam, ab66110) in accordance with the manufacturer’s instructions. Nuclei were counterstained with diamidino-2-phenylindole (DAPI; 1:1000 in HBSS). Cells were observed and imaged using an epifluorescence microscope (AxioObserverA1; Zeiss).

### Quantitative real-time reverse transcription polymerase chain reaction (qPCR)

Cells were homogenized by TriReagent®, and total RNA was isolated using the phenol–chloroform method as previously described by Chomczynski and Sacchi [34]. cDNA synthesis and qPCR were performed as described previously [26] using Tetro cDNA Synthesis Kit (Bioline) and SensiFast SYBR & Fluorescein Kit (Bioline) respectively. Primer concentration in each reaction equalled to 500 nM and their sequences are listed in Table 1. The average fold change in the gene expression of experimental cultures was compared with control cultures and calculated by the 2−DDCt method in relation to the housekeeping gene—GAPDH.

**Table 1:** Primer sequences

Gene	Primer	Sequence 5′-3′	Amplicon length (bp)
p53	F:	TACTCCCCTGCCCTCAACAA	252
	R:	AGGAATCAGGGCCTTGAGGA	
BAX	F:	GCCAGCAAATTGGTGCTCAA	260
	R:	AGCAGTCACTTCCATGGCTC	
BCL-2	F:	TTCTTTGAGTTCGGTGGGGT	164
	R:	GGGCCGTACAGTTCCACAA	
GAPDH	F:	GATGCCCCAATGTTTGTGA	250
	R:	AAGCAGGGATGATGTTCTGG	

*p53* tumour suppressor p53; *BAX* Bcl-2-associated X protein; *BCL-2* B cell lymphoma 2; *GAPDH* glyceraldehyde-3-phosphate dehydrogenase

### Case description

**Fig. 1:**
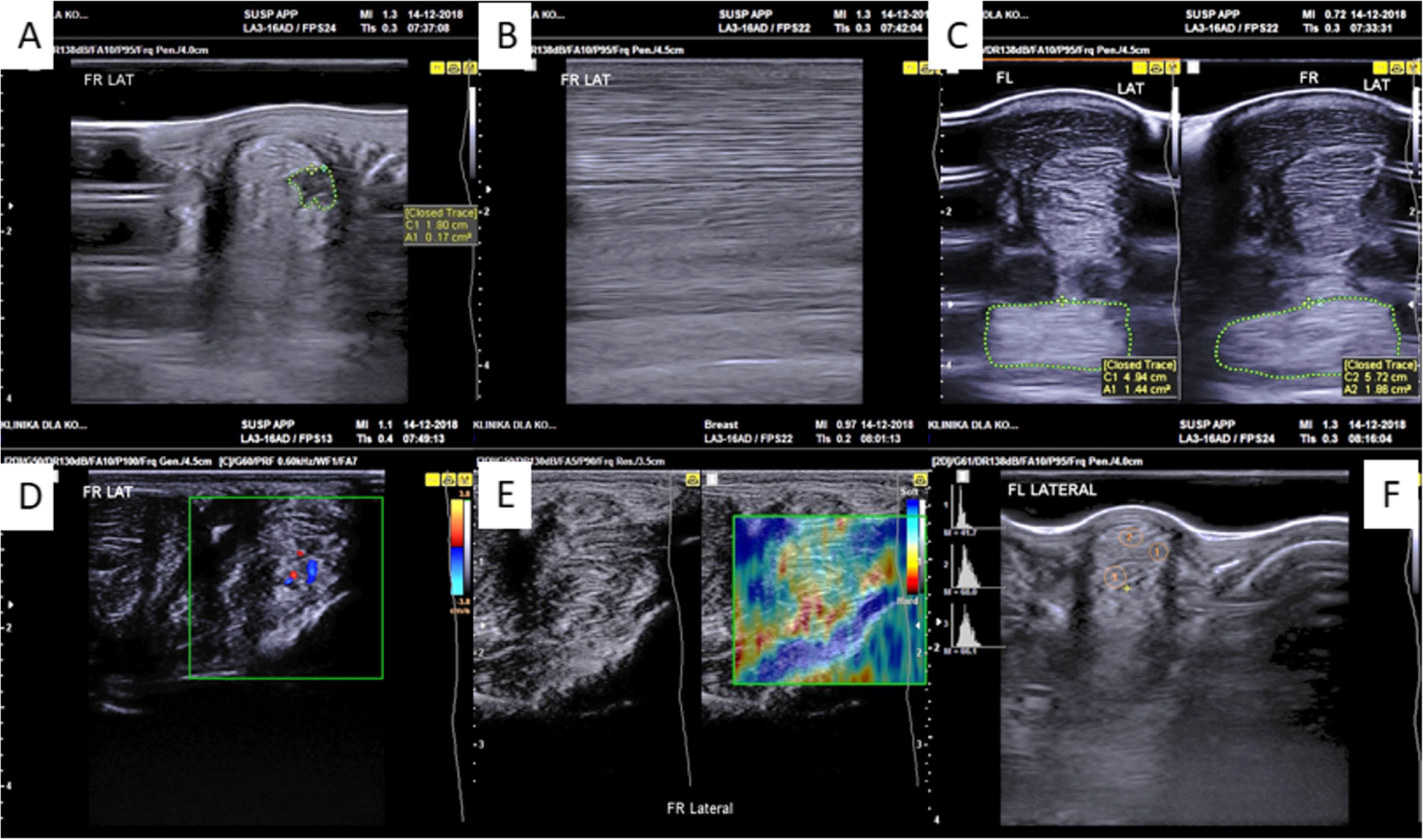
Injury of SL imaged by USG. **a** Transverse image of the SL injury in B1 area. **b** Longitudinal image of the injury. **c** Comparison of SL between left (health) and right (with injury) forelimbs. Decreased echogenicity and increased area in the transverse section are visible. **d** Increased angiogenesis in the injury site. **e** RTE showing diverse elasticity in the injury site. **f** Histograms showing decreased value in the injury area (1) in comparison to healthy tissue (2, 3)

**Fig. 2:**
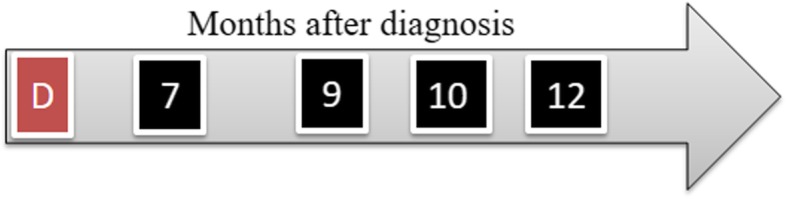
Timeframe of the study. D, diagnosis of SL in jury in right forelimb, week after MVs_AZA/RES_ injection; 7, horse back to training; 9, swelling noted in the proximal-lateral side of the right forelimb in the middle of the cannon, second MVsAZA/RES injection; 10, first clinical evaluation; 12, second clinical evaluation

The study was performed after an approval by the Local Ethics Committee in Wroclaw, Poland (84/2018). The patient was a 6-year-old Dutch Warmblood gelding, working in jumping (up to 1.1 m). The animal was diagnosed with lameness caused by suspensory ligament injury in the right forelimb. Six days after the accident, ultrasonography (USG), including histogram analysis, real-time elastography (RTE), and Doppler ultrasound measurements (SamsungHM70) of both forelimbs, was performed to evaluate the injured region. A complete examination of the SL was performed during each ultrasound examination with both transverse and longitudinal scans. Prior to thermography (Flir T335), the skin in the injury area was shaved. Seven days after injury, a USG-guided injection of MVs_AZA/RES_ directly into the injury site was performed. After 9 months of the first injection, in the same time, swelling in the proximal-lateral side of the right forelimb in the middle of the cannon was also noted. The horse was injected the second time with MVs_AZA/RES_. The first clinical evaluation was performed after 10th while the second clinical evaluation after 12th month of the first MV_AZA/RES_ injection. The injury of SL is shown in Fig. 1 while the timeframe of the experiment in Fig. 2.

### Statistics

All experiments were performed at least in three replicates. Differences between experimental groups were estimated using the un-paired *t* test (Prism5.04; GraphPad Software, La Jolla, CA, USA). Data normality was assessed in all groups by the Shapiro–Wilk test. Differences with probability of *p* < 0.05 were considered significant. Results were presented as mean and standard deviation (SD).

## Results

### MVs_AZA/RES_ exert beneficial effects on ASC_EMS_

**Fig. 3:**
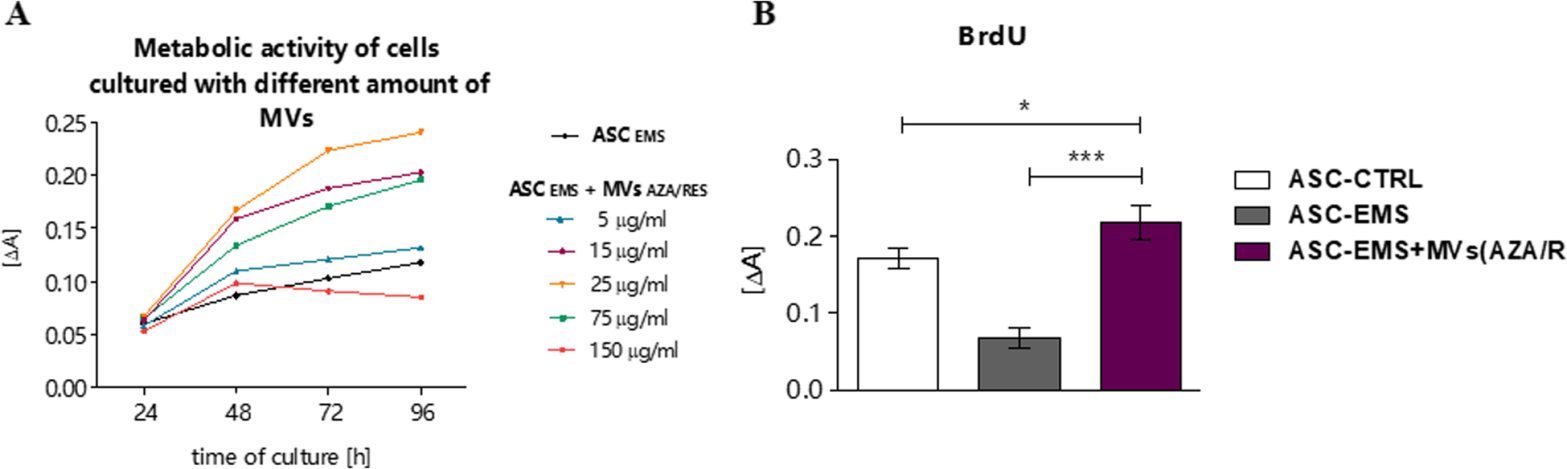
MVs derived from AZA/RES-treated ASC enhance ASC_EMS_ proliferation. Different dosages of MVs were added to ASC in order to investigate whether MVs work in a dose-dependent manner (**a**). The first measurement was performed after 24 h of co-culture. After that time, the medium was exchanged and cell proliferation was monitored till 96 h of culture. For further experiments, cells were cultured for 24 with 25 μg/ml of MVs. BrdU analysis confirmed that selected concentration enhances cellular proliferation. Results are expressed as mean ± SD, *p < 0.05; ***p < 0.001

Different concentrations of MVs_AZA/RES_ were tested in the co-culture with ASC isolated from EMS individuals (Fig. 3a). For further experiments, the most beneficial concentration, 25 μg/ml, was applied. BrdU assay (Fig. 3b) confirmed that MVs_AZA/RES_ enhance ASC proliferation.

### MVs_AZA/RES_ exert anti-apoptotic effects on ASC_EMS_

**Fig. 4:**
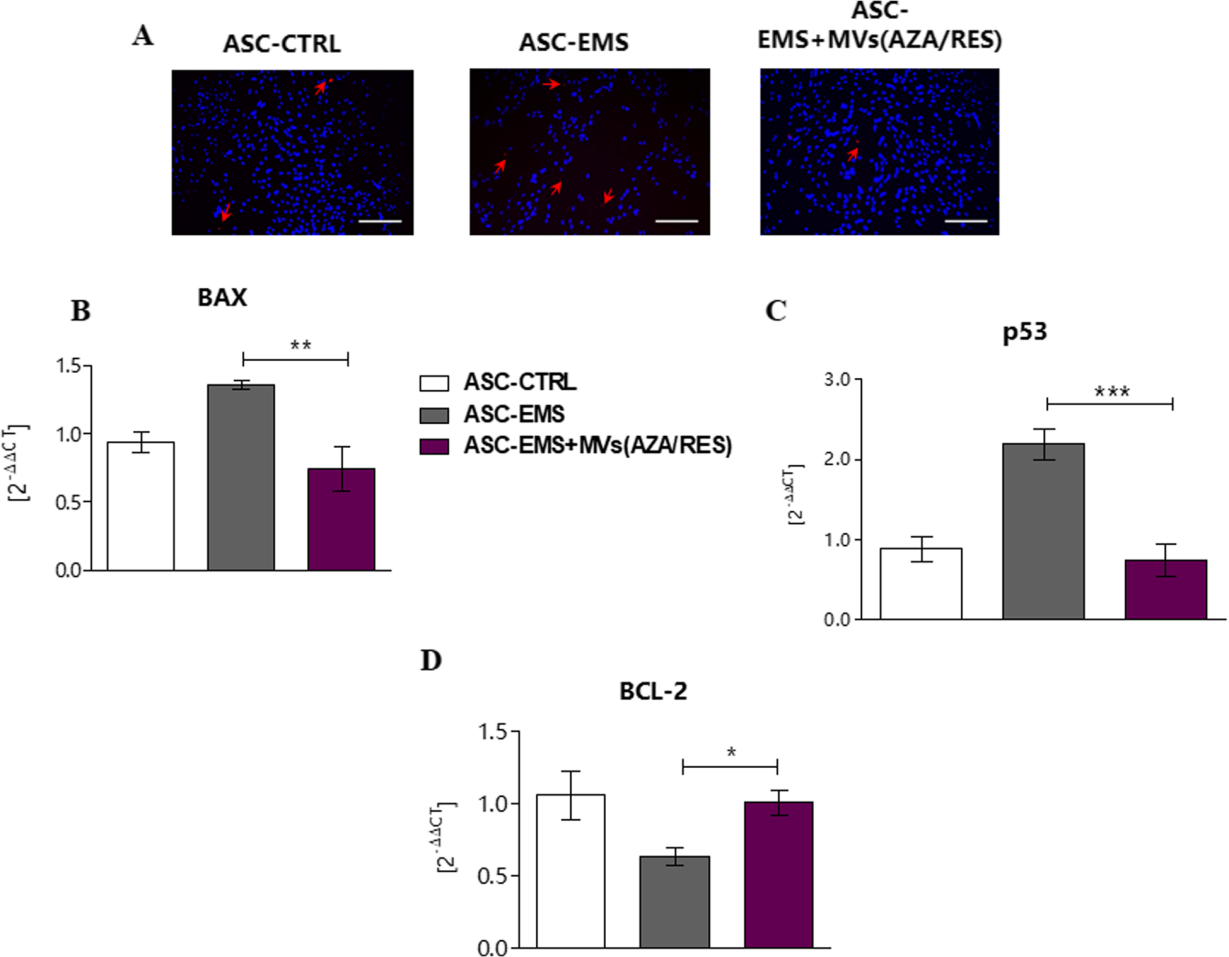
MVs derived from AZA/RES-treated ASC decrease apoptosis in ASC_EMS_. TUNEL staining revealed that MV treatment decreased the number of dead cells in ASC_EMS_ (A). qPCR results revealed that the expression of pro-apoptotic genes BAX (**b**) and p53 (**c**) was decreased in ASC_EMS_ cultured in the presence of MVs. On the other hand, the expression of anti-apoptotic BCL-2 (**d**) was enhanced. Results are expressed as mean ± SD, *p < 0.05; **p < 0.01; ***p < 0.001

TUNEL staining (Fig. 4a) revealed that MVs_AZA/RES_ reduced the number of dead cells. What is more, MV_AZA/RES_ treatment reduced the expression of BAX (Fig. 4b) and p53 (Fig. 4c) but enhanced the expression of anti-apoptotic BCL-2 (Fig. 4d).

### Clinical evaluation after MV_AZA/RES_ injection

**Fig. 5:**
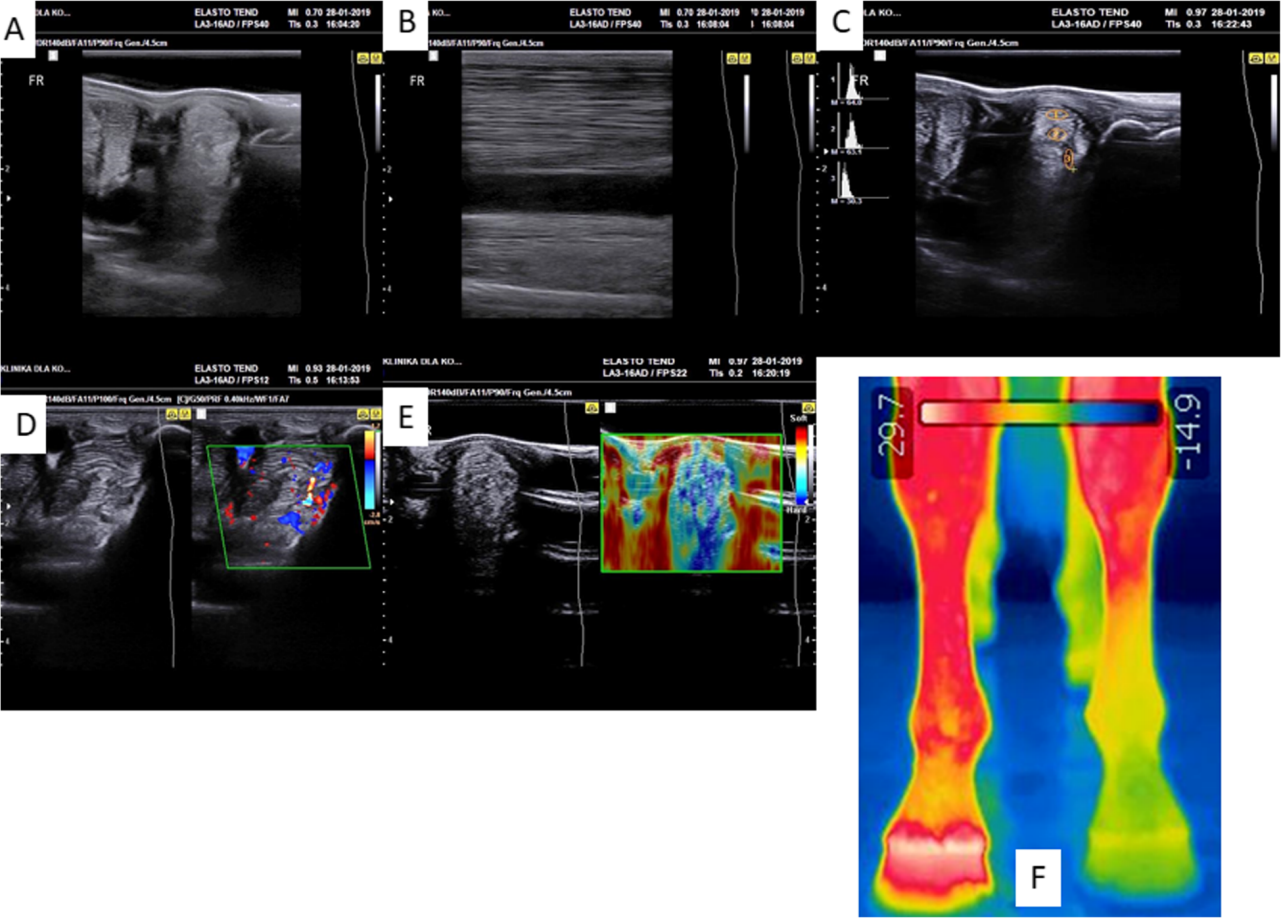
Clinical evaluation after the first MV_AZA/RES_ injection. The resolution of a focal lesion within the SL of the right forelimb. **a** Initiation of lesion filling—transverse image. **b** Lesion filling on a longitudinal image. **c** Increased histogram value in the injury site. **d** Transverse image of enhanced vascularization. **e** Decreased elasticity of the injury site resembling surrounding tissues. **f** Increased temperature in the right forelimb indicates an enhanced angiogenesis after MV injection

Following the injection, the horse showed any adverse reactions. The first examination demonstrated an early clinical improvement in the horse. Initiation of lesion filing with fibrous tissue was observed in transverse (Fig. 5a) longitudinal (Fig. 5b) images. An increased histogram value was observed in the injury site (Fig. 5c). An enhanced vascularization (Fig. 5d) and decreased elasticity (Fig. 5e) of the injury site resembling surrounding tissues were observed. Thermography revealed that an increased temperature in the right forelimb indicates an enhanced angiogenesis after MV injection (Fig. 5f).

**Fig. 6:**
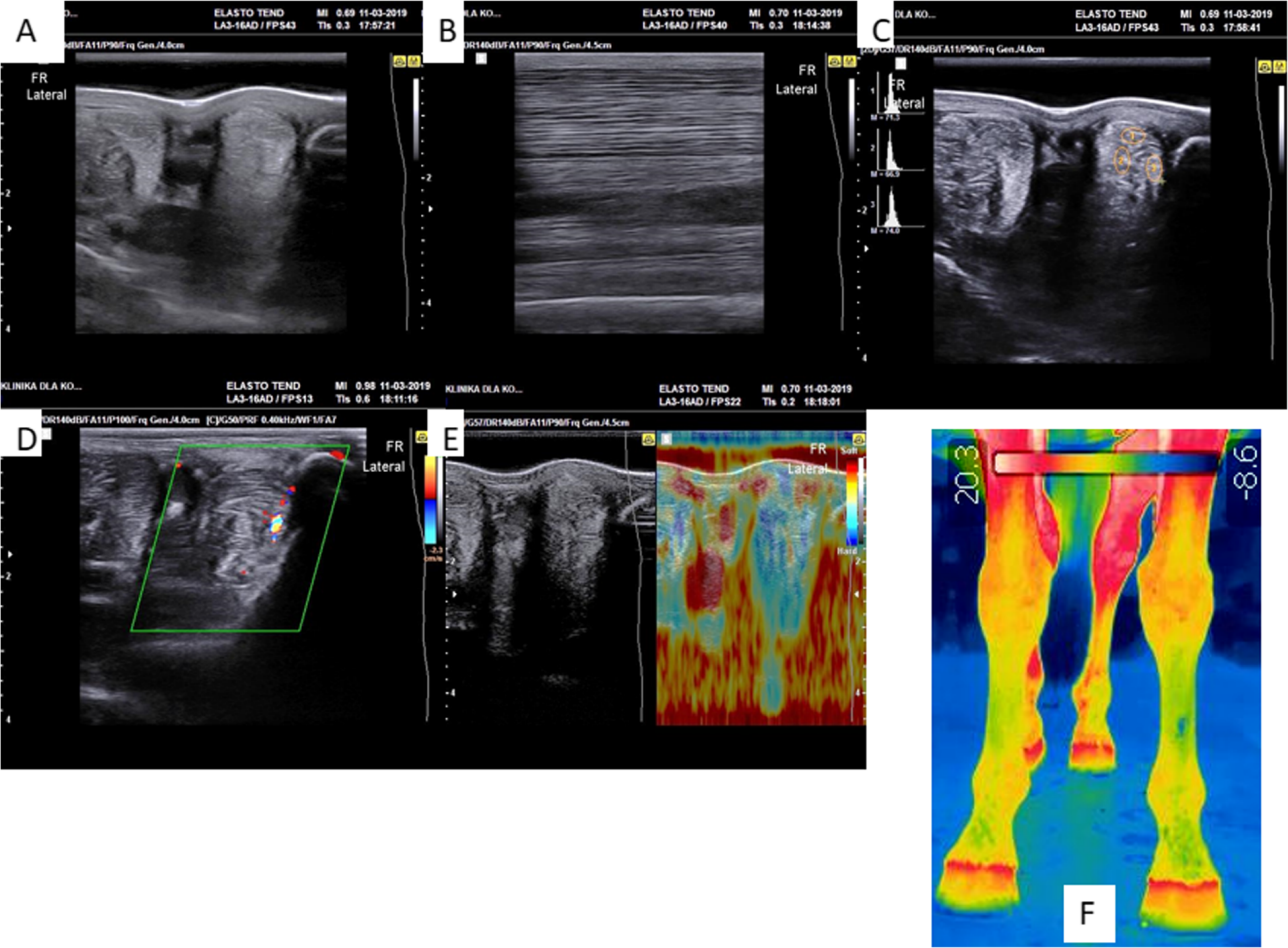
Clinical evaluation after the second MV_AZA/RES_ injection. **a** Transverse images of the injured SL site with a slightly increased echogenicity; the formation of scar tissue separated from surrounding tissues with hypoechogenic fragments can be observed. **b** Longitudinal image. **c** Histograms of varying values between healthy and injured tissue. **d** Moderate blood supply in SL. **e** Moderate differences of elasticity in SL. **f** Thermography showed no pathological changes

In the second examination, transverse images of the injured SL site (Fig. 6a) revealed slightly increased echogenicity and formation of scar tissue separated from surrounding tissues with hypoechogenic fragments. Lesion filling on longitudinal image (Fig. 6b) and increased histograms of varying values between healthy and injured tissue (Fig. 6c) in the injury site were observed as well. Moderate blood supply in SL (Fig. 6d) and moderate differences of elasticity (Fig. 6e) were observed. Thermography showed no pathological changes in the injury site (Fig. 6f).

## Discussion

Multiple reports, including our own, have indicated that MSC therapeutic potential depends on several factors, including patients’ age, lifestyle and health condition [23, 24, 35–37]. Our group has revealed for the first time that in horses diagnosed with equine metabolic syndrome (EMS), isolated ASC suffers from great impairment of their cytophysiological properties [32]. EMS is characterized by obesity, hyperinsulinemia and insulin resistance and frequently leads to the development of laminitis. The main cause of disease is a high-starch diet combined with the lack of physical activity. We have shown that, in vitro culture, ASC from EMS individuals displayed decreased proliferation rate, increased apoptosis and senescence together with mitochondria deterioration [32]. In our previous research, we have demonstrated that autophagy is a rescue mechanism in those cells allowing them to maintain at least a part of their “stemness” [26, 38]. That fact has a profound effect on regenerative medicine which utilizes stem cells for the treatment of different disorders including musculoskeletal injuries. As autologous stem cell therapies are still most desirable, searching for innovative methods able to rejuvenate impaired cells before therapy is strongly recommended. Since now, it was showed that pre-incubation of ASC with certain chemicals, including algae extracts, vitamins and basic fibroblast growth factors, exerts beneficial effects on cell metabolism [24, 39–41]. In our recent research, we have demonstrated that the combination of AZA and RES rejuvenate EMS derived ASC by inhibition of mitochondrial fission [27, 29]. Treated cells were characterized by an increased proliferation rate, reduce apoptosis and senescence, improvement of mitochondria metabolism and enhanced secretion of MVs. In the presented study, we decided to investigate the therapeutic potential of MVs derived from these rejuvenated cells for the treatment of muscle injury in a horse. As MVs are immune privileged, we decided to perform allogenic therapy, using MVs obtained from our previous experiments—MVs were harvested from ASC isolated from EMS individuals treated in vitro with AZA/RES. Our hypothesis was that similar to rejuvenated ASC, their MVs will be characterized by enhanced bioactivity.

As MVs were shown to mimic the beneficial effects of MSC, they are thought to be at least partially responsible for their regenerative properties. In vitro experiments revealed that MVs derived from AZA/RES-treated cells stimulated the proliferation rate of ASC in co-culture in a dose-dependent manner. Similarly, Bruno et al. revealed that MSC-derived MVs increased the proliferation rate of tubular epithelial cells after in vitro injury [42]. What is more, they decreased apoptosis in EMS derived ASC. TUNEL staining indicated that MV treatment reduced the number of dead cells in culture, while qPCR results revealed a decreased expression of pro-apoptotic genes, e.g. p53 and BAX, while anti-apoptotic gene Bcl-2 expression was enhanced. In a cisplatin-induced lethal model of acute kidney injury, several injections of MVs derived from MSC increased anti-apoptotic gene expression, including Bcl-2, in tubular epithelial cells [43]. Similar protective effects of MVs were shown in a model of renal ischaemia/reperfusion injury where MVs inhibited apoptosis and stimulated cellular proliferation [44]. On the other hand, Herrera et al. demonstrated that in human and rat hepatocytes, MVs enhanced proliferation and decreased apoptosis through mRNA shuttled into recipient cells [45].

Due to their frequent and demanding physical activity, sport horses suffer from great mechanical overload of musculoskeletal system which makes them especially prone for traumatization and injuries. Especially common is injury of the suspensory ligament (SL) which contributes to pain and lameness. Since now, regenerative properties of MVs were mainly studied in different animal models of tissue injury [42, 44]. In the presented study, we decided to inject MVs from rejuvenated ASC into injured suspensory ligament in order to decrease inflammation, enhance angiogenesis and trigger a regenerative response of resident cells. Previous studies as a therapeutic approach for SL injuries utilized platelet-rich plasma (PRP), cellular bone marrow or tenogenically induced allogeneic peripheral blood mesenchymal stem cells [10, 46]. In the study performed by Vandenberghe et al. [47], allogeneic tenogenically induced peripheral blood-derived MSCs combined with PRP were utilized for the treatment of a proximal SL injury with positive outcome. On the other hand, studies performed in vitro revealed that PRP and acellular bone marrow (ACB) increased the expression of cartilage oligomeric matrix protein (COMP) production in equine suspensory ligament fibroblasts (SLF) [46]. As equine ABM and PRP are rich in anabolic factors that promote matrix synthesis, their application to injured ligaments is also justified. Our report for the first time demonstrated the application of MSC-derived MVs for SL injury treatment. The obtained results correspond with the data obtained in the research mentioned above. SL ligament is a complex structure as it is composed of soft tissue and bone components. In the presented report, diagnosis was made by USG, Doppler, RTE and thermography. A strong limitation of our study is the lack of angiography which could bring more information about lesion severity. As MSCs were shown to form ectopic bone in the calcified area [48], the application of MVs for SL injury seems to be a safe and reasonable alternative. The first application of MVs resulted in an initiation of lesion filling with scar tissue, increased parallel fibre pattern and moderate diffuse decrease in echogenicity. What is more, we observed an increased temperature in the area of lesion which correlated with the increased angiogenesis in the injury site.

In recent years, regenerative medicine has focused on soluble factors released by MSC, including MVs. As MVs mimic several of the biological actions exerted by MSC, they have become an alternative for stem cell-based therapies. Here, we proved for the first time the safe and effective application of MVs isolated from AZA/RES-treated cells for the treatment of suspensory ligament in athletic horse. Still, further research needs to be performed in order to fully understand the mechanism and therapeutic potential of these bioactive factors.

## Conclusions

Here, we presented that MVs_AZA/RES_ exert rejuvenating effects in vitro as they enhanced cellular proliferation and decreased apoptosis. Beneficial effects of MVs_AZA/RES_ were confirmed in vivo for the treatment of SL injury in horse, as enhanced vascularization and healing were observed.

## Data Availability

The datasets generated during and/or analysed during the current study are available from the corresponding author on reasonable request.

## Abbreviations

ACB: Acellular bone marrow
ASCs: Adipose-derived stem cells
AZA: 5-Azacytidine
bFGF: Basic fibroblast growth factors
CCL-2: Chemokine (C-C motif) ligand 2
COMP: Increased expression of cartilage oligomeric matrix protein
EMS: Equine metabolic syndrome
HGF: Hepatocyte growth factor
IGF-1: Insulin-like growth factor 1
IL-6: Interleukin 6 (IL-6)
miRNA: MicroRNA
mRNA: Messenger RNA
MSCs: Mesenchymal stem cells
MSC-EMS: MSC isolated from equine metabolic syndrome diagnosed animals
MVs: Microvesicles
MVs_AZA/RES_: Microvesicles derived from 5-azacytydine and resveratrol adipose-derived stem cells
PRP: Platelet-rich plasma
RES: Resveratrol
SL: Suspensory ligament
SLF: Equine suspensory ligament fibroblasts
USG: Ultrasonography
VEGF: Vascular endothelial growth factor
